# One Health surveillance—A cross-sectoral detection, characterization, and notification of foodborne pathogens

**DOI:** 10.3389/fpubh.2023.1129083

**Published:** 2023-03-08

**Authors:** Elina Tast Lahti, Nadja Karamehmedovic, Hilde Riedel, Linnea Blom, Jeppe Boel, Elisabetta Delibato, Martine Denis, Alieda van Essen-Zandbergen, Aurora Garcia-Fernandez, Rene Hendriksen, Anna Heydecke, Angela H. A. M. van Hoek, Tom Huby, Renata Kwit, Claudia Lucarelli, Karl Lundin, Valeria Michelacci, Slawomir Owczarek, Isaac Ring, Jette Sejer Kjeldgaard, Ingegerd Sjögren, Milena Skóra, Mia Torpdahl, María Ugarte-Ruiz, Kees Veldman, Eleonora Ventola, Magdalena Zajac, Cecilia Jernberg

**Affiliations:** ^1^Department of Epidemiology and Disease Control, National Veterinary Institute (SVA), Uppsala, Sweden; ^2^Department of Microbiology, Public Health Agency of Sweden, Solna, Sweden; ^3^Department of Biology, Swedish Food Agency, Uppsala, Sweden; ^4^Department of Bacteria, Parasites and Fungi, Statens Serum Institut (SSI), Copenhagen, Denmark; ^5^Department of Food Safety, Nutrition and Veterinary Public Health, Istituto Superiore di Sanità (ISS), Rome, Italy; ^6^Research Unit of Hygiene and Quality of Poultry and Pork Products, French Agency for Food, Environmental and Occupational Health and Safety (ANSES), Ploufragan, France; ^7^Department of Bacteriology, Host-Pathogen Interaction, and Diagnostics Development, Wageningen Bioveterinary Research (WBVR) Part of Wageningen University and Research (WUR), Lelystad, Netherlands; ^8^Department of Infectious Diseases, Istituto Superiore di Sanità (ISS), Rome, Italy; ^9^Technical University of Denmark, The National Food Institute (DTU Food), Copenhagen, Denmark; ^10^Center for Research and Development, Uppsala University/Region Gävleborg, Gävle, Sweden; ^11^Centre for Infectious Disease Control (Department Zoonoses and Environmental Microbiology), Dutch National Institute for Public Health and the Environment (RIVM), Bilthoven, Netherlands; ^12^Animal and Plant Health Agency (APHA), Weybridge, United Kingdom; ^13^Department of Microbiology, National Veterinary Research Institute (PIWet), Pulawy, Poland; ^14^Clinical Microbiology, Uppsala University Hospital, Uppsala, Sweden; ^15^Clinical Microbiology, Region Halland, Halmstad, Sweden; ^16^VISAVET Health Surveillance Centre, Universidad Complutense Madrid, Madrid, Spain

**Keywords:** One Health surveillance, External Quality Assessment, proficiency tests, detection and characterization, notification, foodborne pathogens

## Abstract

**Introduction:**

Several Proficiency Test (PT) or External Quality Assessment (EQA) schemes are currently available for assessing the ability of laboratories to detect and characterize enteropathogenic bacteria, but they are usually targeting one sector, covering either public health, food safety or animal health. In addition to sector-specific PTs/EQAs for detection, cross-sectoral panels would be useful for assessment of the capacity to detect and characterize foodborne pathogens in a One Health (OH) perspective and further improving food safety and interpretation of cross-sectoral surveillance data. The aims of the study were to assess the cross-sectoral capability of European public health, animal health and food safety laboratories to detect, characterize and notify findings of the foodborne pathogens *Campylobacter* spp., *Salmonella* spp. and *Yersinia enterocolitica*, and to develop recommendations for future cross-sectoral PTs and EQAs within OH. The PT/EQA scheme developed within this study consisted of a test panel of five samples, designed to represent a theoretical outbreak scenario.

**Methods:**

A total of 15 laboratories from animal health, public health and food safety sectors were enrolled in eight countries: Denmark, France, Italy, the Netherlands, Poland, Spain, Sweden, and the United Kingdom. The laboratories analyzed the samples according to the methods used in the laboratory and reported the target organisms at species level, and if applicable, serovar for *Salmonella* and bioserotype for *Yersinia*.

**Results:**

All 15 laboratories analyzed the samples for *Salmonella*, 13 for *Campylobacter* and 11 for *Yersinia*. Analytical errors were predominately false negative results. One sample (*S*. Stockholm and *Y. enterocolitica* O:3/BT4) with lower concentrations of target organisms was especially challenging, resulting in six out of seven false negative results. These findings were associated with laboratories using smaller sample sizes and not using enrichment methods. Detection of *Salmonella* was most commonly mandatory to notify within the three sectors in the eight countries participating in the pilot whereas findings of Campylobacter and *Y. enterocolitica* were notifiable from human samples, but less commonly from animal and food samples.

**Discussion:**

The results of the pilot PT/EQA conducted in this study confirmed the possibility to apply a cross-sectoral approach for assessment of the joint OH capacity to detect and characterize foodborne pathogens.

## 1. Introduction

One Health (OH) is a concept often defined as an integrated, unifying approach to sustainably balance and optimize the health of people, animals and ecosystems (1, 2). OH recognizes the health of humans, domestic and wild animals, plants and the wider environment are closely linked and interdependent. This OH approach, therefore, calls for collaboration, coordination, communication and capacity building across disciplines, sectors, organizations and national borders in support of complex health challenges (2). Although OH is not a new concept, it was in 2008 adopted as a joint strategy of the World Health Organization (WHO), the Food and Agriculture Organization (FAO) and the World Organization for Animal Health (WOAH, then OIE) (3). To address the European challenges of foodborne zoonoses (FBZ), antimicrobial resistance (AMR) and emerging threats (ET), a 5-year One Health European Joint Programme (OHEJP) was established in 2018 as a partnership between 37 partners across 19 countries in Europe (3). The main focus of the OHEJP is to enhance harmonization of methodologies, databases and procedures for the assessment and management of FBZ, AMR, and ET across Europe. Surveillance of zoonoses and investigations of foodborne and zoonotic outbreaks are examples of OH activities requiring correct diagnostics and sensitive and specific analytical methods across sectors and disciplines.

National, regional, and local authorities, physicians, veterinarians, food business operators and laboratories within animal health, food safety, and public health sectors may have different approaches on when and how to analyze a sample from animals, food, or humans for enteropathogenic *Campylobacter, Salmonella* and/or *Yersinia*. The samples may, for instance, originate from official control or surveillance programmes on animal health or food safety, or from Hazard Analysis Critical Control Point (HACCP) samplings at food companies or be taken from patients in hospitals or from outpatients for determination of an illness or be part of an outbreak investigation (4, 5). The protocols for testing these pathogens may vary, for instance, between sectors, countries, regions, or sample types. In addition, after the laboratory analyses, the findings of the pathogens may have a different legal status regarding whether the finding is mandatory to notify or not to a corresponding authority (4). Thus, these variabilities have an impact on the possibilities to detect, investigate and contain clusters and outbreaks and thus impose control or preventive measures.

Also, collection and interpretation of data across sectors and countries can be challenging in a OH perspective. Thereby, the context of the data collected and reported needs to be known to correctly evaluate the results. Other tools to improve the comparability of data between the sectors and countries are, to a certain degree, to harmonize laboratory methods and/or testing the capacity for detection and characterization of the relevant pathogens independently of the laboratory methods used.

According to Zoonoses Directive 2003/99, all Member States in the European Union (EU) shall collect relevant data on zoonoses and zoonotic agents in primary production and/or at other stages in the food chain. *Campylobacter* and *Salmonella* are among the zoonotic agents to be included in monitoring, whereas *Yersinia* is to be covered according to the epidemiological situation. Also, Member States shall investigate foodborne outbreaks. Data collected within the monitoring programmes and investigations of foodborne outbreaks shall be reported to the European Food Safety Authority (EFSA) but data from other samplings may not be collected. However, as only part of the monitoring is harmonized, results from the national monitoring programmes are difficult to compare (4).

On EU level within the public health sector, notifications of campylobacteriosis, salmonellosis and yersiniosis are mandatory in most Member States (4). In some countries, notifications can also be based on a voluntary system. The EU case definitions https://eur-lex.europa.eu/legal-content/EN/TXT/PDF/?uri=CELEX:32018D0945&from=EN#page=12 for the diseases are updated regularly taking into account, e.g., developments of diagnostic techniques. The case definitions on national level and the capacity of detecting a case can, however, differ between countries, for reasons which could often be attributed to other factors rather than the diagnostic capacity of the laboratories. The number of reported cases generally underestimates the true number or cases (6, 7). Underestimation may occur when asymptomatic cases or cases with mild symptoms do not seek health care, medical care does not test cases or not notify them (8).

Proficiency testing (PT) is, according to ISO 17043:2010, defined as an evaluation of participant performance against pre-established criteria by means of interlaboratory comparisons. The PT schemes can, for instance, be qualitative, quantitative, or sequential in nature. The term External Quality Assessment (EQA) is more often used in the medical field as a synonym for PTs, but EQAs can also be designed to provide insight into the complete path of workflow of the laboratory, and not just the testing processes. A common feature in EQA programmes is education of participants. Some EQA programmes are compulsory, either required by an accrediting body or by law whereas others are voluntary, and the quality manager may choose to voluntarily participate in an EQA programme (https://www.who.int/publications/m/item/overview-of-external-quality-assessment-eqa). Participation in PT or EQA schemes is pivotal for assessment of the performance of the laboratory and identification of potential problems.

There are national, EU-wide, and international sector-specific PT and EQA schemes designed in a quality-assured manner for assessing the ability to detect, identify and characterize enteropathogenic bacteria, especially for *Campylobacter* and *Salmonella* and to a certain extent for *Yersinia* (9, 10). The EU Reference Laboratories (EURL) of food, feed and animal health appointed by the European Commission are obliged to annually organize PTs to the National Reference Laboratories of Member States (10, 11). Likewise, EQAs are routinely organized for the national public health laboratories on characterization but not on detection of these pathogens (10, 12, 13). PTs/EQAs are also offered by national and international commercial quality assurance panel providers. Metagenomics-based cross-sectoral or sector-specific PTs involving viruses (14–16), parasites (17), and recently also bacteria have been organized (18). However, joint cross-sectoral panels for detection of foodborne pathogens from simulated samples are, to the authors knowledge, currently lacking.

The pilot PT/EQA aimed at assessing the cross-sectoral capacity of European laboratories to detect, characterize and notify three defined zoonotic foodborne bacteria and developing recommendations for future cross-sectoral PTs/EQAs. Detection in this study refers to the diagnostic test, i.e., the analysis steps identifying the target pathogen whereas characterization refers to species, (bio)serotype (BT) and sequence type (ST) determination. Notification in this study is defined as reporting of a finding of a pathogen to the responsible authorities. The specific objective was to prepare simulated samples to resemble matrices (samples) analyzed at animal health, food safety and public health laboratories. Public health laboratories in this study refer to clinical microbiological laboratories (primary laboratories) and national public health laboratories. The laboratories were also asked to describe if findings of these pathogens were mandatory notifiable according to their national legislation or guidelines.

## 2. Materials and methods

### 2.1. Outline of the PT/EQA

The participants of the pilot PT/EQA were recruited among the partner institutions of the OHEJP CARE “Cross-sectoral framework for Quality Assurance Resources for countries in the European Union” project (*n* = 12) in eight countries including Denmark, France, Italy, the Netherlands, Poland, Spain, Sweden, and the United Kingdom. Additionally, three public health laboratories participated in the pilot from one of the partner countries. Of the 15 participating laboratories, five represented public health, four food safety, two animal health, three both food safety and animal health and one laboratory covered both public health and food safety. These categorisations are based on the information the participants reported.

The participants received a fictive scenario of a foodborne outbreak among persons hunting wild boar and visiting a small-scale abattoir (Supplementary material 1). The dispatched samples were to simulate stool samples from diseased patients, environmental samples from food-producing premises or fecal samples from animals. The participants were assigned to analyze the samples for detection of *Campylobacter, Salmonella* and *Yersinia* using the detection and characterization methods and practices applied at the laboratory. They were also requested to identify the target bacteria at a species level and include information of the serovar for *Salmonella* and bioserotype for *Yersinia* if the participants had methods available for these characterisations.

### 2.2. Production and quality control of the PT/EQA

Each participant received five samples containing 35 mL of matrix simulating a sample and five vials containing freeze-dried bacteria, designated Care 1-5, hereinafter referred to as C1-5 (Table 1). The concentrations of the target bacteria varied between 4.1 x 10^4^ and 3.7 x 10^5^ colony-forming units (cfu). Before analyzing the samples, the vials with freeze-dried bacteria were to be dissolved with 1 mL of sterile diluent and transferred to the matrix.

**Table 1:** Mean of concentration (m), index of dispersion (I_2_) and reproducibility (T) values from the quality control of the target organisms.

**Vial^a^**	**Target organisms**	**Analysis^b^**	**Mean^c^**	**I_2_^e^**	**T^f^**
C1	*C. coli*	mCCDA, 37°C, 48 h	3.7 x 10^5^	8.1	1.9
C2	*S*. Stockholm	BHI agar, 37°C, 24 h	4.1 x 10^4^^d^	2.1	1.6
C2	*Y. enterocolitica* O:3/BT4	BHI agar, 37°C, 24 h	9.9 x 10^4d^	0.8	1.3
C3	*S*. Enteritidis	BHI agar, 37°C, 24 h	6.0 x 10^4d^	0.6	1.2
C3	*C. jejuni*	mCCDA, 37°C, 48 h	5.6 x 10^4^	0.5	1.2
C5	*Y. enterocolitica* BT1A	BHI agar, 37°C, 24 h	1.2 x 10^5^	-	-

a Five vials of C1, C3 and C4 and ten vials of C2 and C5 were analyzed in duplicate.

b mCCDA, Modified Charcoal Cephoperazone Deoxycholate Agar; d BHI, Brain Heart Infusion.

c Concentration mean in cfu/mL.

d From analysis of a parallel sample mixture.

e Index of dispersion.

f Test of reproducibility.

*Yersinia enterocolitica* O:3/biotype 4 and biotype 1A are hereinafter abbreviated to O:3/BT4 and BT1A, respectively. Vials C1-4 were freeze-dried in portions of 0.5 mL (19) using Epsilon 1-12 D (Christ, Osterode, Germany). Vials C5 were freeze-dried using an ALPHA 1-4/LD plus (Christ, Osterode, Germany) in portions of 1 mL.

Quality control of C1-C4 was performed on ten randomly selected vials in conjunction with manufacturing or on five vials if the sample mixture was already approved for homogeneity. Homogeneity of a sample mixture was approved if the values obtained for the test of reproducibility (T) and the test index of dispersion between vials (I_2_) did not simultaneously exceed 2.6 and 2.0, respectively (20, 21).

The sample labeled “Matrix” represented an environmental sample from an abattoir or a stool sample or a composite environmental sample from wild boars, i.e., all laboratories received the same matrix composition. The matrix was prepared by dissolving 0.5 kg of autoclaved pig manure in 4 L sterilized buffered peptone water (BPW) (Oxoid LP0034, Basingstoke, UK) with NaCl (Merck 6404, Rahway, NJ, USA), mixed by swirling and then stored at +4°C overnight. The following day, the solution was decanted and autoclaved at +134°C for 45 min. The matrix was stored at +4°C until use.

Quality controls of the matrix were performed with cultivation methods and biochemical tests to analyze if *Campylobacter jejuni, C. coli, Salmonella* spp. or *Y. enterocolitica* were present above the detection limit that could influence the participants' downstream results. In addition, the presence of *Salmonella* spp. was analyzed using MicroSEQTM *Salmonella* detection kit (Thermo Fisher Scientific, Waltham, MA, USA). The cultivation methods and biochemical tests used to examine the matrix were performed according to the following methods from the Nordic Committee on Food Analysis (NMKL No. 119 3rd ed. 2007, NMKL No. 71 5th ed. 1999, NMKL No. 117 3rd ed. 1996). *C. jejuni, C. coli, Salmonella* spp. or *Y. enterocolitica* were not present above the detection limit in the matrix. The matrix was also tested on the BD MAX™ System (BD Diagnostics, Hunt Valley, MD, USA), a fully automated extraction and real-time PCR machine, using the BD MAX™ Enteric Bacterial Panel and BD MAX™ Extended Enteric Bacterial Panel at a public health laboratory. The BD MAX™ System was not able to detect *Campylobacter, Salmonella* or *Yersinia* spp. in the matrix.

### 2.3. Methods for characterization of the target organisms by the PT/EQA providers

All target organisms were characterized using whole genome sequencing (WGS). Automated nucleic acid extraction and purification were performed with PSS MagLEAD 12 gC (Precision System Science Co., Ltd, Chiba, Japan) and DNA concentration (ng/μL) was quantified with Qubit^®^ 2.0 Fluorometer (Thermo Fisher Scientific). The Ion Xpress^TM^ Plus Fragment Library Kit for AB Library Builder™ System (Thermo Fisher Scientific) was used for library preparation and Ion S5 XL system (Thermo Fisher Scientific) for sequencing. An additional sequencing of the samples was performed using Illumina NovaSeq 6000 (Illumina, Inc., San Diego, CA, USA), and carried out at the Clinical Genomics, Science for Life Laboratory, Stockholm, Sweden.

Quality trimming and assembly of the genome were performed with CLC Assembly Cell software (version 5.2.0.; Qiagen, Denmark) using the settings (clc_quality_trim –c-25 and clc_assembler -v -q -o). Species was identified by BLAST toward an in-house database with reference sequences (22) and sequence type (ST) was determined using the Multi Locus Sequence Typing (MLST) scheme from PubMLST for *Campylobacter* (23), the MLST scheme from Enterobase for *Salmonella* (24–26) and the Enterobase McNally MLST scheme for *Yersinia* (26).

*In silico* serovar prediction for *Salmonella* was performed with an in-house database of STs and corresponding serovars in combination with SeqSero (27).

### 2.4. Distribution of the PT/EQA

The participating laboratories were informed on 5 January 2021 *via* email about the anticipated number of samples and approximate time point (month) for the PT/EQA.

Samples were dispatched under refrigeration by a courier in accordance with the International Air Transport Association (IATA) packing instructions 650 for UN3373, on 12 April 2021. All 15 participants received five vials, five matrix samples, a temperature logging device, instructions, and a material safety data sheet.

### 2.5. Questionnaire

Instructions and a personal link for reporting were sent by email to the contact person(s) at each laboratory. The laboratories were instructed to initiate the analyses the same week the PT/EQA was received. The participants were requested to report their results *via* a web-based questionnaire at the latest on 31 May 2021. In addition to questions on the results of the pilot PT/EQA, the web-based questionnaire included questions on the laboratory methods applied, on notification practices as well as the type of samples the laboratories usually receive (Supplementary material 2).

## 3. Results

### 3.1. Quality control

Sequencing using the IonTorrent and Illumina platforms yielded the same result except for one sample (Table 2). Analysis of the Illumina sequence data of *Y. enterocolitica* O:3/BT4 of C2 showed that the virulence factors YadA (*Yersinia* adhesin A), VirF, and the Yops (*Yersinia* outer proteins) were missing, while being present in the Ion Torrent sequence data, suggesting that the *Yersinia* ~70-kb virulence plasmid (pYV), encoding the virulence factors, may have been lost. The genome size for the Illumina sequence data showed a smaller genome compared to the Ion Torrent genome size, indicating a plasmid loss. The extracted DNA for the Illumina sequencing were from an additional cultivation cycling.

**Table 2:** Microorganisms present in the vials. Target organisms are characterized with whole genome sequencing and indicated in bold font.

**Vial**	**Microorganisms**	**Reference^a^**	**Sequence type (ST)**
C1	***Campylobacter coli**, Citrobacter freundii, Escherichia coli* O157 (*stx* neg) and *Listeria monocytogenes*	CCUG 45147	ST860
C2	***Salmonella*** **Stockholm**, ***Yersinia enterocolitica*** **O:3/BT4**, *Escherichia coli* and *Klebsiella rhizophila*	SLV-390, CCUG 45643	ST3214, ST276
C3	***Salmonella*** **Enteritidis** ***Campylobacter jejuni** Escherichia coli* and *Staphylococcus saprophyticus*	SLV-436, SLV-540	ST11, ST21
C4	*Micrococcus* sp., *Klebsiella oxytoca, Escherichia coli, Staphylococcus aureus, Enterococcus faecalis, Bacillus cereus, Candida* spp. and *Clostridium perfringens*		
C5	***Yersinia enterocolitica*** **BT1A**	CCUG 46850	ST147

a Culture collection. CCUG, Culture Collection University of Gothenburg, Sweden; SLV, Swedish Food Agency.

### 3.2. Arrival of the PT/EQA and start of the analysis

The participants received the pilot PT/EQA on 13 April (13 participants) and 14 April 2021 (2 participants).

The analyses were initiated on 13 April (*n* = 4), 14 April (*n* = 3), 14 and 15 April (*n* = 1), 19 April (*n* = 4), 13 May (*n* = 1), and 15 May (*n* = 1), 2021. One participant initiated the analysis of *Salmonella* on 19 April, the analysis of *Campylobacter* and that of *Yersinia* on 3 May 2021. After arrival, the package was stored at refrigerator temperature (+3–+8°C) at 11 laboratories, in a freezer (−20°C) at two laboratories and at room temperature (+20–+22°C) at two laboratories. The laboratories that stored the package at room temperature initiated the analysis upon arrival.

### 3.3. Detection and characterization of *Campylobacter* spp.

Of the 15 participating laboratories, 13 analyzed the samples for *Campylobacter*. Eight participants performed enrichment prior to plating onto a selective medium. There were some differences between the laboratories whether one or two selective media were used for detection (Appendix Table 1), and whether one or several methods, biochemical tests, Matrix-Assisted Laser Desorption/Ionization- time-of-flight mass spectrometry (MALDI-TOF), microscopy, PCR and WGS were used for species identification (Appendix Table 2). In total, five laboratories used PCR for detection and characterization of *Campylobacter* and one public health laboratory used the commercial real-time PCR system BD MAX™ (BD Molecular Diagnostics). The amount of the sample used for detection varied between 10 μL and 10 mL, the public health laboratories used smaller sample sizes (Appendix Table 1).

*Campylobacter* spp. were present in two vials, *C. coli* in C1 and *C. jejuni* in C3. Of the laboratories testing for *Campylobacter*, all 13 correctly detected the target organism in C1 (Table 3). Eleven laboratories reported the result at species level (*C. coli*), one at genus level and one as either *C. jejuni* or *C. coli*. Twelve of the laboratories testing for *Campylobacter* reported a correct detection result for C3. Ten laboratories reported the result at species level (*C. jejuni*), one at genus level and one as either *C. jejuni* or *C. coli*.

**Table 3:** Results of the PT/EQA reported by the participants.

**Lab code**	**Vial**								
	**C1**	**C2**	**C2**	**C3**	**C3**	**C4**	**C5**	**False negative results**	**False positive results**
L1	*C. coli*	*S*. Stockholm	*Y. enterocolitica* O:3/BT4	*S*. Enteritidis	*C. jejuni*	No target microbes	*Y. enterocolitica* BT1A	0	0
L2	*C. coli*	*S*. Stockholm	*Y. enterocolitica*	*S*. Enteritidis	*C. jejuni*	ND	*Y. enterocolitica*	0	0
L3	*C. coli*	*S*. Stockholm	*Y. enterocolitica* O:3/BT4	*S*. Enteritidis	ND	ND	*Y. enterocolitica* BT1A	1	0
L4	*C. coli*	*S*. Stockholm	ND	*S*. Enteritidis	*C. jejuni*	ND	*Y. enterocolitica* BT1A	1	0
L5	*C. coli*	*S*. Stockholm	NA	*S*. Enteritidis	*C. jejuni*	ND	NA	0	0
L6	*C. coli*	*S*. Stockholm	*Y. enterocolitica* O:3	*Salmonella* spp.	*C. jejuni*	ND	*Y. enterocolitica* non-pathogenic	0	0
L7	*C. coli*	ND	ND	*S*. Enteritidis	*C. jejuni*	ND	*Y. enterocolitica* BT1A	2	0
L8	*C. coli/jejuni*	*Salmonella* spp.	ND	*S*. Enteritidis	*C. coli/jejuni*	ND	*Y. enterocolitica* non-pathogenic	1	0
L9	NA	*S*. Stockholm	NA	*S*. Enteritidis	NA	ND	NA	0	0
L10	*C. coli*	*S*. Stockholm	*Y. enterocolitica* BT4	*S*. Enteritidis	*C. jejuni*	ND	*Y. enterocolitica* BT1A	0	0
L11	*C. coli*	*Salmonella* spp.	*Y. enterocolitica*	*Salmonella* spp.	*C. jejuni*	ND	*Y. enterocolitica*	0	0
L12	*C. coli*	*Salmonella* spp.	*Y. enterocolitica*	*Salmonella* spp.	*C. jejuni*	ND	*Y. enterocolitica* non-pathogenic	0	0
L13	*Campylobacter* spp.	ND	ND	*Salmonella* spp.	*Campylobacter* sp.	ND	*Y. enterocolitica Campylobacter* spp.	2	1
L14	*C. coli*	*Salmonella* spp.	NA	*Salmonella* spp.	*C. jejuni*	*Salmonella* spp.	NA	0	1
L15	NA	*S*. Stockholm	NA	*S*. Enteritidis	NA	ND	NA	0	0

NA, not analyzed; ND, not detected.

One false negative result was reported for sample C3 and one false positive result of *Campylobacter* spp. for sample C5. Two different laboratories reported these results and the laboratory reporting the false negative result for sample C3 correctly detected *Campylobacter* in sample C1.

### 3.4. Detection and characterization of *Salmonella* spp.

All fifteen participating laboratories analyzed the samples for *Salmonella*. Nine laboratories performed both pre-enrichment and enrichment prior to plating onto selective media (Appendix Table 3). Five of the six laboratories not performing pre-enrichment belonged to the public health sector and two of them did not use any enrichment methods. The amount of the sample used for detection varied between 10 μL and 25 mL, the public health laboratories using smaller sample sizes.

Species identification was performed using one or several methods: biochemical tests, MALDI-TOF, PCR and WGS (Appendix Table 4). Two public health laboratories used PCR for detection of *Salmonella*, one of them the commercial real-time PCR system BD MAX™.

Most laboratories performing serotyping of *Salmonella* used conventional slide agglutination according to the White-Kauffmann-Le Minor scheme. Three laboratories used WGS for species identification, *in silico* serovar and ST determination, either as a primary method or in addition to the other methods.

*Salmonella* spp. was present in two vials, *Salmonella* Stockholm in C2 and *Salmonella* Enteritidis in C3. Two public health laboratories reported false negative results for *S*. Stockholm and were the only laboratories that did not use enrichment methods. The other laboratories detected *Salmonella* and nine of them reported serovar Stockholm. All laboratories detected *Salmonella* in sample C3 and ten laboratories reported serovar Enteritidis (Table 3). One false positive result for *Salmonella* was reported for sample C4.

### 3.5. Detection and characterization of *Y. enterocolitica*

Of the 15 participating laboratories, eleven analyzed the samples for *Yersinia* spp. Six laboratories used enrichment methods prior to plating onto a selective medium (Appendix Table 5). The laboratories not using any enrichment methods were from the public health sector. The amount of the sample used for detection varied between 10 μL and 25 mL, the public health laboratories using smaller sample sizes.

Species and bioserotype identifications were performed by one or several methods: biochemical tests, MALDI-TOF, PCR and WGS (Appendix Table 6). Three public health laboratories used PCR for detection of *Y. enterocolitica*, one of them used real-time PCR and one the commercial real-time PCR system BD MAX™.

The target organism *Y. enterocolitica* was present in two vials: O:3/BT4 in C2 and BT1A in C5 (Table 2). Seven of the eleven participating laboratories correctly identified *Y. enterocolitica* in sample C2. Four of the laboratories reported the results at a bioserotype or serotype level, correctly assigning O:3/BT4 or O:3 (Table 3). False negative results were reported by four public health laboratories not using enrichment methods in their routine methodology.

All eleven laboratories testing for *Yersinia* spp. identified *Y. enterocolitica* in sample C5, however, one of the laboratories obtained deviating results, reporting both *Y. enterocolitica* and *Campylobacter* spp. in the sample. Five of the eleven laboratories correctly reported BT1A.

### 3.6. Accreditation status of the participating laboratories

Of the 15 participants, all, except one, were accredited or quality assured for detection of *Salmonella*, eleven for detection of *Campylobacter* and seven for *Yersinia*. Five of the six public health laboratories were accredited or quality assured for detection of all the three target pathogens. Of the 11 laboratories accredited or quality assured for detection of *Campylobacter*, five covered public health, three food safety, two animal health and one both animal health and food safety. Five of the six laboratories accredited or quality assured for detection of *Yersinia* covered public health and one food safety. No animal health laboratory was accredited or quality assured for detection of *Yersinia*.

### 3.7. Notification of *Campylobacter* spp., *Salmonella* spp. and *Y. enterocolitica*

Notifications of findings of *Salmonella* in human samples were mandatory in six countries (Denmark, Italy, Poland, Spain, Sweden, and the UK) and in two countries (France and the Netherlands) notifications were based on a voluntary system (Table 4). Notification of *Salmonella* in food samples was mandatory in seven countries whereas conditional in animal samples in five of the eight countries. Notifications could depend on the serovar or whether the sampling was performed within official monitoring programmes. Notifications of findings of *Campylobacter* from human samples were mandatory for five countries (Denmark, Poland, Spain, Sweden, and the UK) and in three countries (France, Italy, and the Netherlands) notifications were based on a voluntary system. Notification in animal and food samples could depend on the animal species and/or matrix or whether the sampling was performed within official monitoring programmes. Detection of *Yersinia* was rarely notifiable in animal and food samples. In human samples notifications of yersiniosis were mandatory in five countries (Denmark, Poland, Spain, Sweden, and the UK), notifications based on a voluntary system in two countries (France and Italy), whereas the Netherlands has no surveillance system in place for yersiniosis. In two countries BT1A of *Y. enterocolitica* was excluded from the case definition. Two of the public health laboratories indicated that no pathogenic *Yersinia* was detected in sample C5, which was correct according to the notification criteria for these participants, since the target bacterium was *Y. enterocolitica* BT1A.

**Table 4:** Notification status of the findings of *Campylobacter, Salmonella*, and *Yersinia enterocolitica* within animal health, food safety and public health of the participating countries.

**Country**	***Campylobacter*** **spp**.			***Salmonella enterica spp***.			***Yersinia enterocolitica***		
	**Animals**	**Foods**	**Humans**	**Animals**	**Foods**	**Humans**	**Animals**	**Foods**	**Humans**
Denmark	No	No	Mandatory	Yes	Yes	Mandatory	No	No	Mandatory
France	No	No	Voluntary	Conditional^c^	Yes	Voluntary	No	No	Voluntary
Italy	Conditional^a^	Conditional^a^	Voluntary	Yes	Yes	Mandatory	Conditional^a^	Conditional^a^	Voluntary
Netherlands	No	No	Voluntary	Conditional^d^	Yes	Voluntary	No	No	No
Poland	No	Yes	Mandatory	Conditional^a^	Yes	Mandatory	No	No	Mandatory
Spain	Conditional^a^	Conditional^a^	Mandatory	Conditional^a^	Conditional^a^	Mandatory	Conditional^a^	Conditional^a^	Mandatory
Sweden	Conditional^b^	No	Mandatory	Yes	Yes	Mandatory	No	No	Mandatory
UK	No	No	Mandatory	Conditional^e^	Yes	Mandatory	No	No	Mandatory

a Notifiable if the sampling was performed within official monitoring programmes.

b Only findings in poultry are notifiable.

c Mandatory notification of serovars Typhimurium (and the monophasic variant), Enteritidis, Infantis, Virchow, Hadar.

d Mandatory notification of serovars Typhimurium (and the monophasic variant) and Enteritidis.

e Mandatory notification if detected from livestock.

### 3.8. Detection or characterization of *Campylobacter* spp., *Salmonella* spp. and *Y. enterocolitica* from routine samples

Of the participants, all but two replied that they routinely received samples for detection of *Salmonella*, 11 for testing of *Campylobacter* and five for *Yersinia* (Table 5). Twelve participants received isolates of *Salmonella* for further characterization, eight for *Campylobacter* and six for *Yersinia*. The four laboratories not analyzing *Y. enterocolitica* routinely belonged to the food or animal health sector.

**Table 5:** Detection and characterization of *Campylobacter, Salmonella*, and *Yersinia enterocolitica* from primary samples or isolates within animal health, food safety and public health of the participating laboratories.

**Lab code**	***Campylobacter***		***Salmonella***		***Yersinia***		**Sector**

	**Detection**	**Characterization**	**Detection**	**Characterization**	**Detection**	**Characterization**	
L1	Yes	Yes	Yes	Yes	No	No	F + V
L2	No	No	Yes	Yes	No	No	F + V
L3	No	Yes	No	Yes	No	Yes	F + P
L4	Yes	Yes	Yes	Yes	Yes	Yes	P
L5	Yes	No	Yes	Yes	No	No	V
L6	Yes	Yes	Yes	Yes	Yes	Yes	F
L7	No	Yes	No	Yes	No	Yes	P
L8	Yes	No	Yes	No	Yes	No	P
L9	Yes	Yes	Yes	Yes	No	No	F + V
L10	Yes	No	Yes	Yes	No	No	F
L11	Yes	No	Yes	No	No	No	V
L12	Yes	No	Yes	No	Yes	Yes	P
L13	Yes	Yes	Yes	Yes	Yes	Yes	P
L14	Yes	Yes	Yes	Yes	No	No	F
L15	No	No	Yes	Yes	No	No	F

F, food safety sector; P, public health sector; V, animal health sector.

## 4. Discussion

This pilot PT/EQA is, to the authors' knowledge, the first cross-sectoral PT/EQA organized on detection and characterization of bacterial foodborne pathogens in matrices simulating samples analyzed within public health, animal health and food safety. The aim of this pilot was to assess the joint capacity to detect and characterize the target pathogens by using not specific predefined methods but by using the methods available at the laboratories, i.e., simulate the conditions of investigations of foodborne outbreaks. Molecular methods are more commonly used at primary and reference laboratories and WGS has become an important tool for typing. Genomic data enables more reliable and precise information on source attribution.

All the participants, except one, used accredited or quality assured methods for detection and characterization. Most of the participants detected the target pathogens *Campylobacter, Salmonella* and *Y. enterocolitica* in the samples C1, C2, C3 and C5 of this PT/EQA. Regarding deviating results, most of the reported false negatives, six out of seven, were reported for sample C2 including the target bacteria *Salmonella* Stockholm and *Y. enterocolitica* O:3/BT4. These were concentrated to public health laboratories not using enrichment methods as part of their routine methodology in addition to using smaller sample sizes. The absence of enrichment, a smaller sample size and a lower concentration of target organisms in this sample may explain the observed challenges in detection, especially in a complex background flora as feces. For detection of *Salmonella* in stool samples, enrichment culture was significantly more sensitive than PCR using BD MAX (28). Thus, enrichment could be recommended unless a PCR method is shown as sensitive as the culture method.

The concentration of target bacteria in the vials used in the PT/EQA varied between 4.1 x 10^4^ and 3.7 x 10^5^ cfu/mL. When analyzing food samples or animal samples for asymptomatic carriers for *Campylobacter, Salmonella* or enteropathogenic *Yersinia* the aim is to detect low levels of these bacteria. On the contrary, when clinical samples are analyzed, the detection limit does not need to be as low, due to the higher number of pathogens. Thus, for detection in animal and food matrices by using enrichment methods, the pilot PT/EQA was probably not challenging whereas for public health laboratories not applying an enrichment step, the levels of 10^4^ cfu/mL could be close to the detection limit. However, detection of *Campylobacter* at the same levels was not problematic.

Moreover, two false positive results were reported by different laboratories, one for *Salmonella* and one for *Campylobacter*. These results might have been a result of cross-contamination at the laboratory or a mistake in the reporting phase.

Especially on the public health side, more and more laboratories are changing from culture-based detection methods to PCR-based. Using PCR or other molecular-based methods, test results can be available already after 2–3 h if an enrichment is not applied whereas the culture-based methods can take from one up to several days. Many laboratories do not necessarily proceed further after the PCR step and isolation attempts may be performed only when testing for antimicrobial resistance is needed for treatment, for typing in outbreak investigations, or for targeted surveillance. The PCR results are often enough for notification as a criterium of a laboratory confirmed case, as the EU case definitions show. For detection and characterization of these pathogens from food and animal matrices, according to the EU Control Regulation 2017/625, the use of standard methods is preferable. Alternative methods, such as PCR, are allowed if they are validated against the standard method according to ISO 16140-6:2019.

Three of the public health laboratories used multiplex PCR as the primary detection method, either a commercial system or an in-house method. The BD MAX™ system for enteric pathogens was used by one laboratory without performing any enrichment of the samples. In a study using spiked samples, BD MAX™ system demonstrated 100% sensitivity for *C. jejuni* and *Salmonella* spp. tested at the following concentrations of bacteria in a sample (artificially produced by mixing stool samples with bacteria): 10^7^ cfu/mL, 10^6^ cfu/mL and 10^5^ cfu/mL (29). At 10^4^ cfu/mL the sensitivity of BD MAX™ was 100% for *C. jejuni* but only 69% for *Salmonella* spp. and 44% at 10^3^ cfu/mL, which might explain the difficulties with detecting *Salmonella* spp., but not *Campylobacter* spp. in the pilot PT/EQA.

A poor performance of *Y. enterocolitica* detection and lack of non-*Y. enterocolitica* detection was demonstrated by assessing four commercially available real-time PCR systems, including the BD MAX™ system (30). The poor agreement observed in the study of the four PCR systems for detection of *Y. enterocolitica* might be explained by known heterogeneity between strains and different choices of chromosomal target genes such as *ail*, for detection of pathogenic *Y. enterocolitica*, and *yst*B, which is also present in most BT1A strains (31, 32). The target gene for *Yersinia* in the BD MAX™ system is *invA* which is also present in non-pathogenic *Yersinia*. Some commercial PCR systems use *ail* as the target gene, which will, with few exceptions, exclude *Y. enterocolitica* BT1A. The *ail* gene is also used as the target gene in the international standard ISO/TS 18867 for the detection of pathogenic *Y. enterocolitica* in the samples of the food chain. On the other hand, different PCR methods for detection of *Salmonella* and *Campylobacter*, in general, do not encounter similar issues related to different target genes.

Analysis of sequence data of *Y. enterocolitica* O:3/BT4 from sample C2 derived from Ion Torrent and Illumina showed that virulence factors involved in the pathogenicity of *Y. enterocolitica*, YadA, VirF, and the Yops, carried on a plasmid, were present in the first sequencing data from Ion Torrent and absent in the later sequencing performed using the Illumina platform. These findings suggest that a spontaneous loss of the pYV plasmid, encoding the virulence factors, may have occurred. The use of plasmid markers alone, may therefore not be sufficient for identification of pathogenic *Y. enterocolitica* in diagnostic settings.

In general, rapid detection or exclusion of bacterial gastrointestinal pathogens in human, food and animal samples is highly requested for the patients, the food industry and the animal keepers. However, bacterial isolates are still required for species determination, subtyping and for susceptibility testing. In future, new molecular techniques like metagenomics, probably minimize the need for cultivation of microorganisms for typing purposes, also for fecal samples.

According to the responses from the PT/EQA participants, the notification practices varied between pathogens, sectors and countries. Notification of all these three pathogens was most common within public health. Findings of *Salmonella* were notifiable across sectors although the notification could be conditional, especially within animal health. Findings of *Campylobacter* in animal or food samples were either not notifiable or conditionally notifiable and findings of *Yersinia* in animal samples were rarely notifiable in any of the countries. In a foodborne outbreak investigation, the findings of these pathogens would nevertheless be reported as part of the outbreak investigation. Due to the differences in legal notification practices, it is specifically challenging to compare and interpret surveillance data between sectors where different criteria are set enabling only specific serovars to be notified or notification is only required within specific animal matrices. However, few studies have investigated the compliance to the notification criteria. A clear variation in incidence and notification of *Campylobacter* and *Salmonella* were seen in a British general practice area (8). Whether there are variations in the compliance to the notification criteria in other regions and other sectors, is unclear.

The matrix in the present panel was similar for all the participants and independent of the sector recipient. This matrix was chosen to enable the same conditions regarding inhibitors, homogenization issues, and concentrations of the target pathogens that could influence the detection for the participants. For further studies to consider in the future, another option could be having different matrices, consisting of the same target pathogens if the sensitivity within the specific matrix would be an important aspect to cover.

The panel was set in an epidemiological context of an outbreak scenario. Cross-sectoral panels put in an outbreak scenario should trigger further discussions between the sectors on differences in methods for detection and typing, and notification rules. For future cross-sectoral panels, the results outcome of the panels, methods used, and notification criteria could be discussion topics for cross-sectoral post PT/EQA workshops. This could in turn increase awareness of cross-sectoral differences which need to be taken into consideration when interpreting surveillance data within OH.

The target foodborne zoonotic organisms for future panels could also have specific resistance profiles, which could be part of the testing capacity. Approaches for phenotypic testing of antimicrobial resistance may vary between sectors. In addition, using WGS for predicting antimicrobial resistance and typing has increased during the last years for e.g., *Salmonella* and *Campylobacter* and could be considered as a characterization option in future schemes. WGS for determination of antimicrobial resistance would primarily be used for surveillance purposes (33) and not for assessing treatment regimens as the phenotypic and genotypic methods do not fully correlate.

In conclusion, this pilot PT/EQA showed that a cross-sectoral approach could be used for assessment of the OH capacity to detect and characterize foodborne pathogens. PTs of the food and animal laboratories are often used to test a specific predefined method whereas the EQA schemes of the public health laboratories are most often used to assess the capacity to correctly detect and characterize independent of the applied methods. Cross-sectoral PT/EQA schemes could result in more general recommendations, e.g., on the target genes for PCRs, or on the characterization methods to apply. Moreover, the organization of such comparative testing schemes stimulates collaboration and discussion across laboratories working in different countries and sectors, setting the ground for further development of methodologies applied to face foodborne zoonosis. Future cross-sectoral PT and EQA schemes should include a genomic aspect, for instance by assessing the performance of the analysis of bioinformatics. The pilot showed that the participating laboratories, however working in different countries and sectors obtained a wide level of agreement even if using different methodologies. This information is currently limited and is pivotal for ensuring comparability of results at the EU level, especially when considering scenarios such as outbreak investigations.

## Data availability statement

Original datasets on sequencing data are available in a publicly accessible repository: The original contributions presented in the study are publicly available. This data can be found here: https://www.ebi.ac.uk/ena/browser/home accession number PRJEB57080. The other original contributions presented in the study are included in the article/Supplementary material, further inquiries can be directed to the corresponding author.

## Author contributions

ET, HR, LB, and CJ designed the study and analyzed the results. ET, NK, HR, LB, and CJ drafted the manuscript. All authors collected data, contributed to the interpretation of the results, contributed to the manuscript, and accepted it.
