# Supplementary material for: One Health surveillance—A cross-sectoral detection, characterization, and notification of foodborne pathogens
Source: Front Public Health. 2023 Mar 8;11:1129083. doi: 10.3389/fpubh.2023.1129083 (PMC10034719; doi:10.3389/fpubh.2023.1129083)
Supplement: Supplementary file 1 [file Data_Sheet_1.docx]

Supplementary material 1

Pilot Proficiency Test (PT)/External Quality Assessment (EQA) on detection and characterisation of food-borne pathogens - Instructions

**One Health European Joint Programme (EJP) CARE IA 2.1-WP1-T2**

**Pilot Proficiency Test (PT)/External Quality Assessment (EQA) on detection and characterisation of food-borne pathogens**

**Starting the week no. 15, 12-16 April 2021**

**Data entry is open until 31 May 2021**

**General information**

The objective of this cross-sectorial pilot proficiency test (PT)/ external quality assessment (EQA) is to assess the ability to detect and characterise enteropathogenic *Campylobacter, Salmonella* and *Yersinia* in samples simulated to resemble matrices analysed at food, veterinary and public health laboratories. The aim of this PT/EQA is, together with the W1-T2-ST2 PT on typing and characterisation including whole genome sequencing (WGS) and W1-T2-ST3 PT on outbreak surveillance based on WGS data, to provide recommendations for future cross-sectorial PTs/EQAs. These three pilot PTs/EQAs are organised within the One Health European Joint Programme project CARE https://onehealthejp.eu/jip-care/.

This PT/EQA is voluntary and free of charge. The participants may decide to perform the PT/EQA for one or all of these three pathogens.

The PT/EQA will be dispatched on Monday, April 12, 2021. The PT/EQA should be started the same week as the test is dispatched from the Swedish Food Agency in Uppsala, Sweden. All results shall be reported in a web-based questionnaire at the latest on **May 31, 2021**.

**Information on the fictive epidemiological setting**

Five persons visited their local general practitioner (GP) with symptoms ranging from severe stomach aches to diarrhoea lasting for more than four days. Four of the cases had participated in a weekend event involving hunting of wild boar. Besides hunting, the event included a tour to the neighbouring small abattoir and an all-inclusive castle hotel visit. The fifth case had delivered chicken and vegetables from a nearby farm to the event and had had a quick lunch at the castle. The weekend event hosted 15 guests, where three additional guests had reported mild gastrointestinal symptoms, which did not involve a visit to the GP.

Samples were sent for analysis targeting any of the gastrointestinal bacterial pathogens *Salmonella*, *Yersinia,* and *Campylobacter*.

- **Five stool samples** were sent to a clinical microbiological laboratory, four from the guests and a fifth from the neighbouring chicken farmer.
- **Five environmental samples** were sent to a food safety laboratory were obtained from the castle and abattoir: from floor drains and surfaces.
- **Five composite environmental samples** were sent to a veterinary laboratory from the wild boar baiting area, manure from the abattoir floor and from the neighbouring chicken farm.

**Outline**

Each participant will receive a package containing:

Five samples: each containing a sample labelled Matrix mimicking an environmental sample from an abattoir, or a stool sample or a composite environmental sample from wild boars. Each sample contains 35 ml of matrix. Thus, it is possible to use a test portion of 10 g for the analysis of each of the target organisms.

Five vials coded CARE 1-5 containing freeze-dried bacteria. The vials may contain one to three of the target organisms *Campylobacter*, *Salmonella* and/or *Yersinia* and background flora.

Enclosed with the PT/EQA is a temperature logging device, T-log, which is a small chip in a plastic bag. It is marked with a number which you do *not* need to record.

**Please do not open the plastic bag, send it as soon as possible to**

**National Veterinary Institute (SVA)**

**Linda Svensson**

**ESS**

**751 89 Uppsala**

**Sweden**

You can send it by regular mail in a regular envelope to this address.

On arrival, examine the package to ensure that it has not been damaged during the transport. Record the condition for later report in a web-based questionnaire.

We recommend you analyse the samples according to the methods (cultural or molecular methods) in use at your laboratory. You may also analyse the samples according to another method than your standard method. For *Campylobacter*, please perform a species identification, for *Salmonella* a result of the serovar and for *Yersinia* a biotype, if you have methods available for these characterisations. If you normally do not perform serotyping, species identification or biotyping, please report your results and procedures in the reporting form. Please describe the method(s) in the reporting form.

Please report the results as well as respond to the questions in the report form in the web-based questionnaire at the latest on the **31^st^ of May 2021**. A personal link to the questionnaire will be sent by email in connection with the distribution of the PT/EQA to the contact person.

**Preparation of the vials and the sample**

Prepare the samples from the vials and the samples according to the instructions below.

1. To dissolve the vial with freeze-dried bacteria, remove the cap of the vial: with the arrow on the cap pointing away from you, carefully pull the flip top up and away from you. When the flip top is vertical pull downwards and either to the left or right until the seal separates. Gently remove the cap from the vial and dispose it in a container for sharp/cutting items. Please note this action may cause a distortion of the rubber stopper initiating a release of vacuum.
2. Reconstitute each vial in turn by aseptically adding 1 ml of Buffered Peptone
   Water or Phosphate Buffered Saline Buffer (or 0.9% of physiological saline).
3. Mix the suspension gently but thoroughly, and then transfer the suspension to the tube containing the mimicked sample.
4. Mix the sample and the suspension thoroughly and then continue the
   analyses of the sample according to the protocols routinely used in your laboratory for detection of *Campylobacter, Salmonella* and *Yersinia*.

**We wish you good luck with the pilot PT/EQA!**
