# Supplementary figures and images for: One Health surveillance—A cross-sectoral detection, characterization, and notification of foodborne pathogens
Source: Front Public Health. 2023 Mar 8;11:1129083. doi: 10.3389/fpubh.2023.1129083 (PMC10034719; doi:10.3389/fpubh.2023.1129083)

Supplementary material 2

Questionnaire


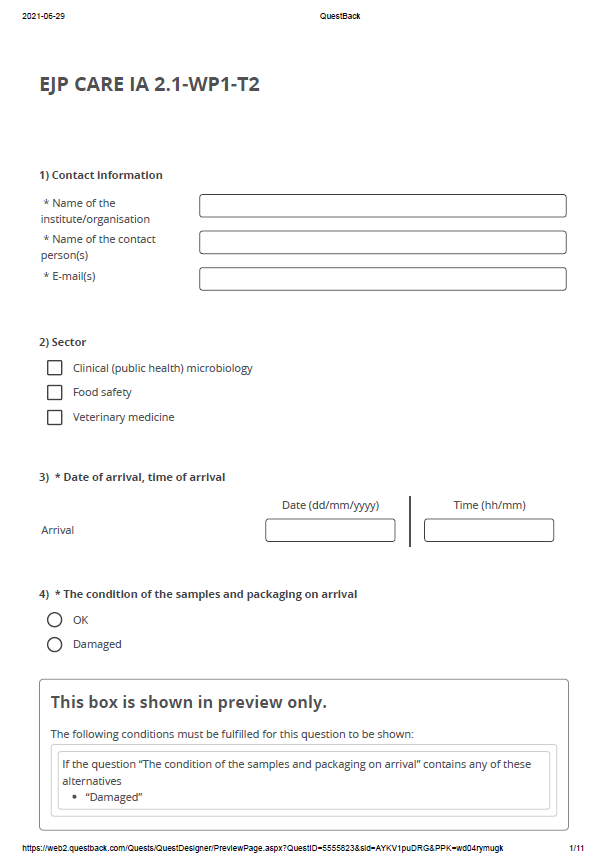


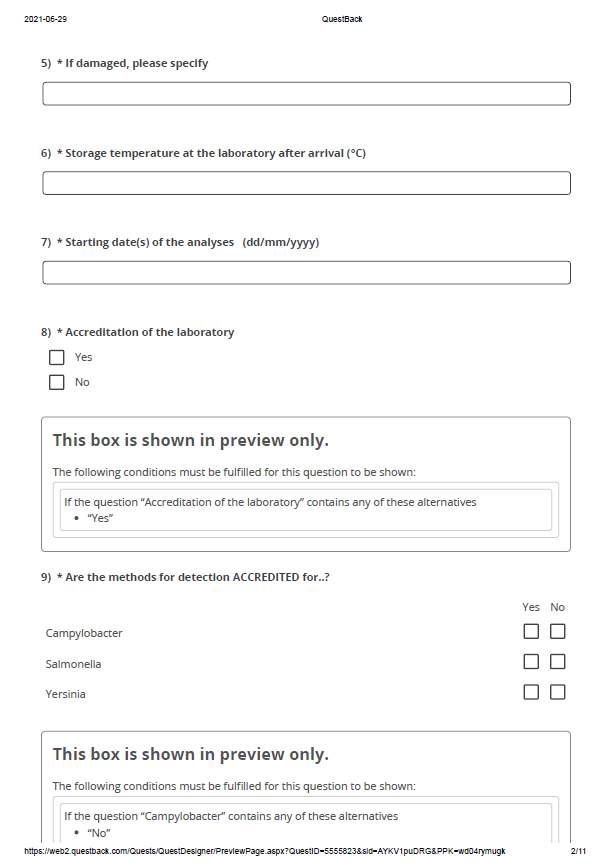


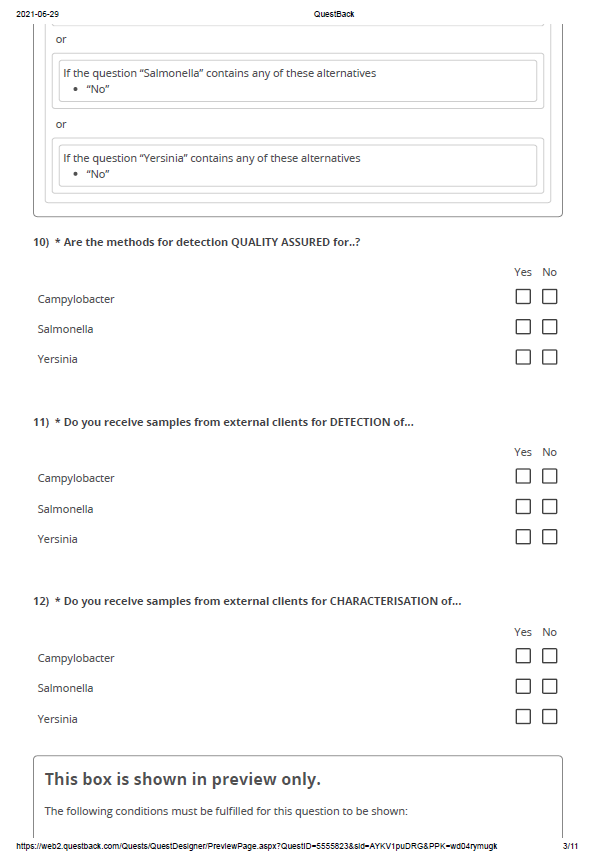


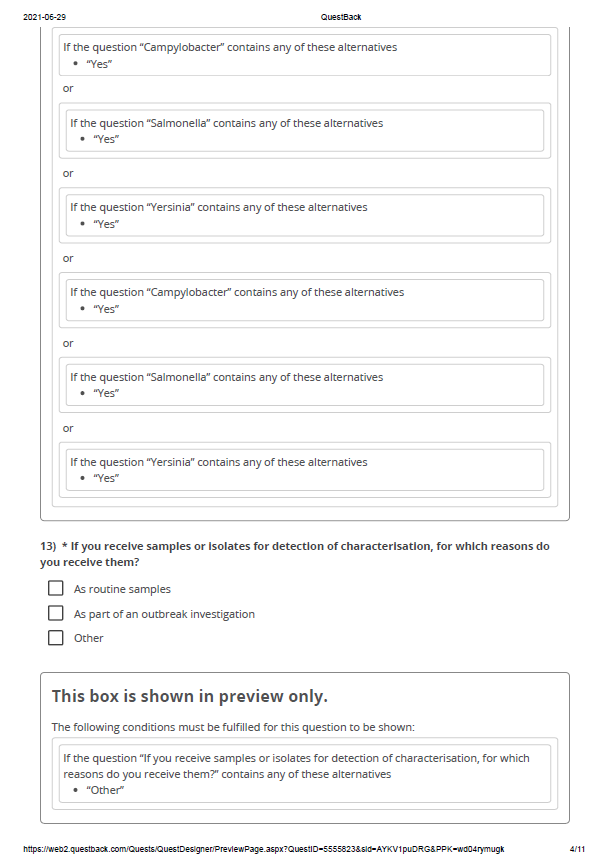


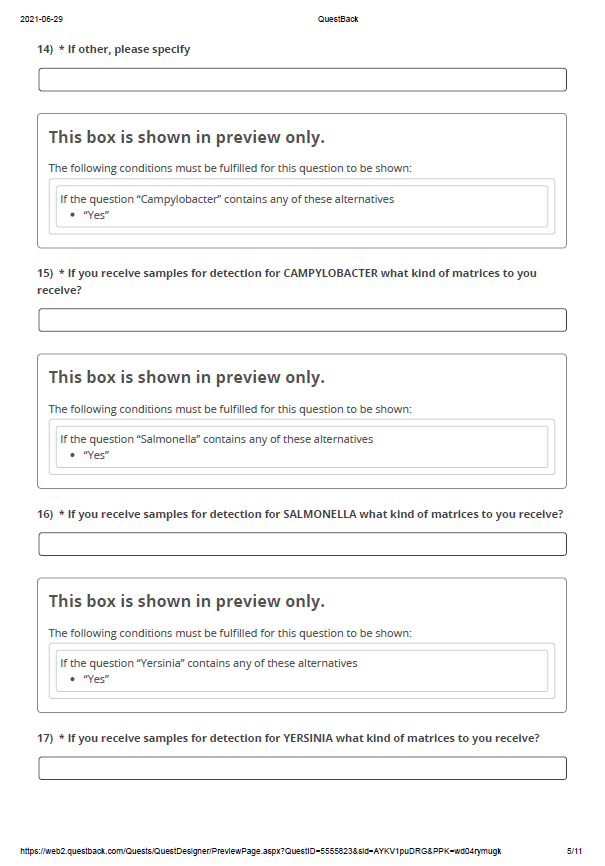


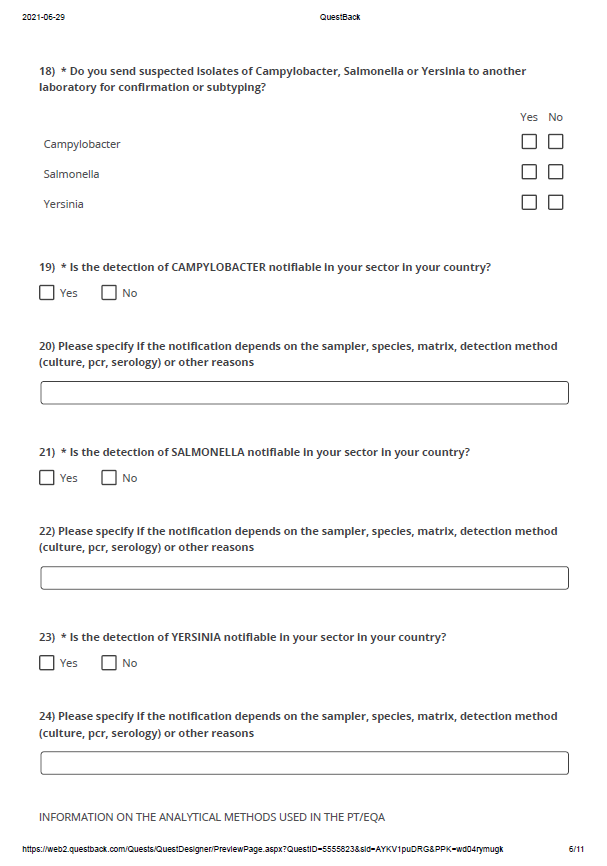


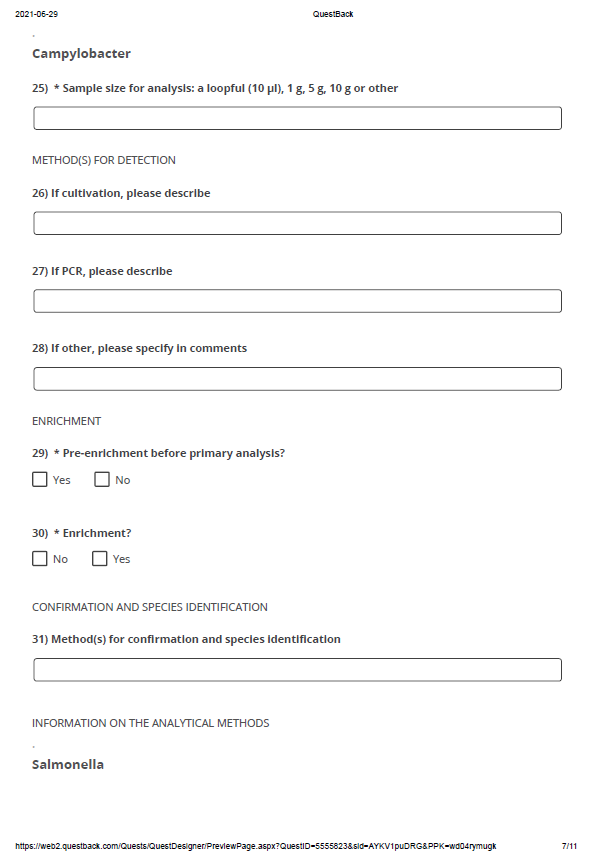


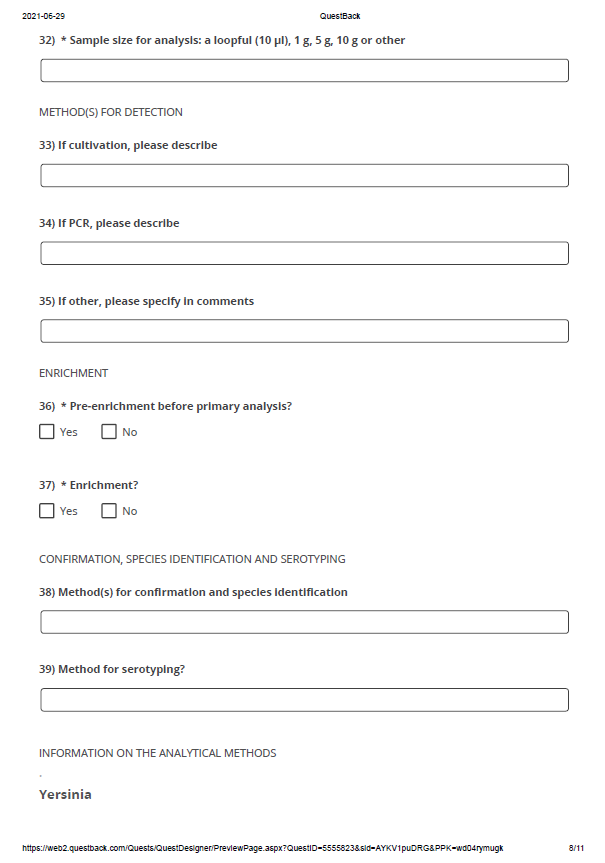


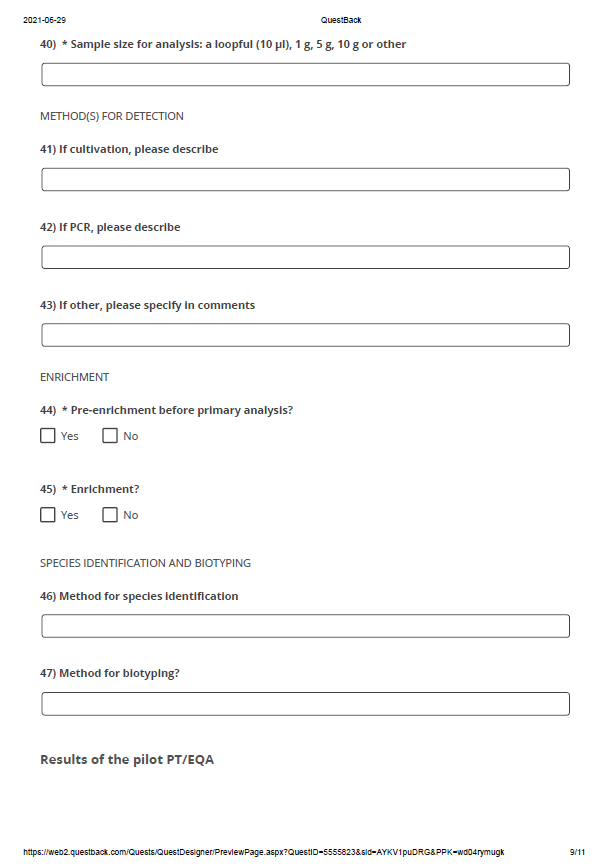


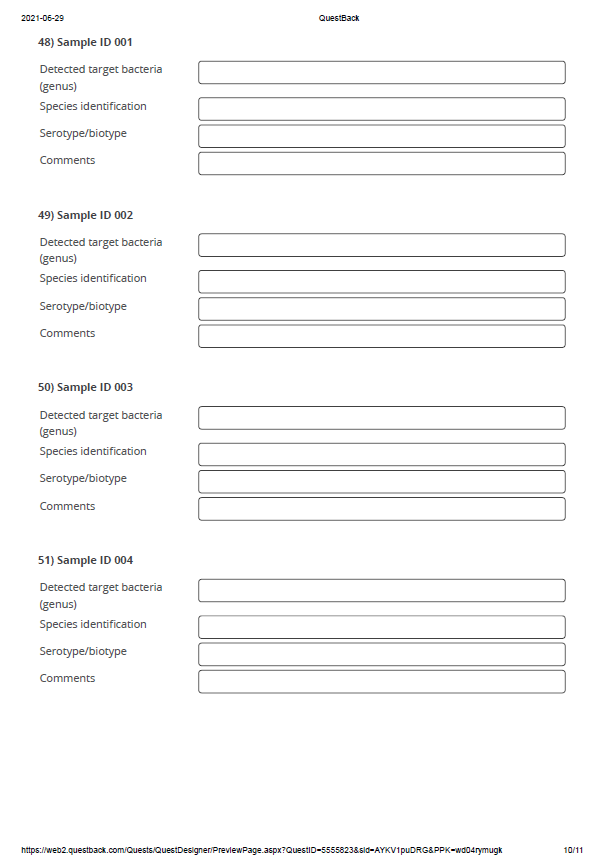


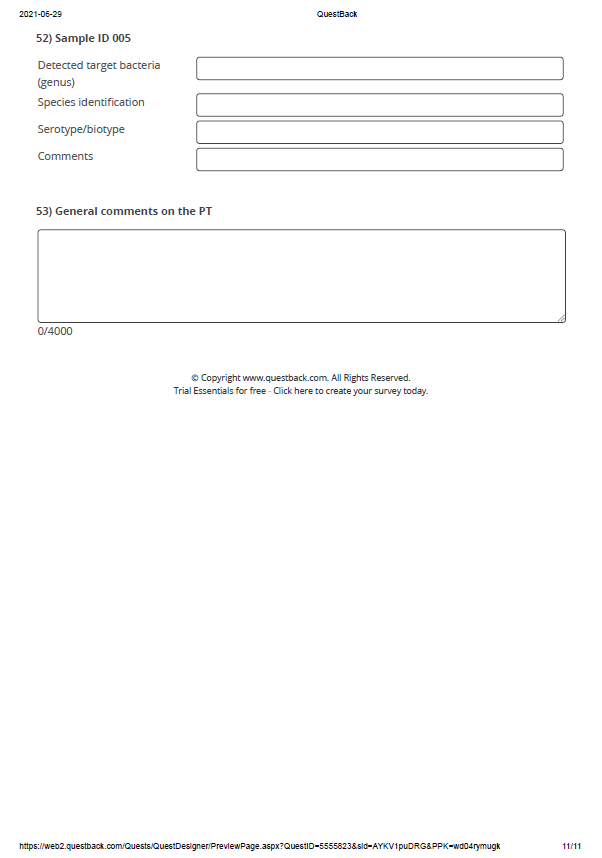

Supplement: Supplementary file 2 [file Data_Sheet_2.docx]
