# Supplementary material for: One Health surveillance—A cross-sectoral detection, characterization, and notification of foodborne pathogens
Source: Front Public Health. 2023 Mar 8;11:1129083. doi: 10.3389/fpubh.2023.1129083 (PMC10034719; doi:10.3389/fpubh.2023.1129083)
Supplement: Supplementary file 3 [file Data_Sheet_3.pdf]

Supplementary material 3

Appendix table 1. Sample size, enrichment and selective medium used for detection of *Campylobacter* spp.

Lab code	Sector	Sample size	Enrichment				Selective medium				
			Yes/No	Medium	Temperature	Period	Medium1	Medium2	Temperature	Period	Atmosphere
L1	F + V	10 mL**	No				mCCDA		41.5°C	48 h	Microaerophilic
L2	F + V	10 mL	Yes	Bolton	41.5°C	48 h	mCCDA	Karmali	41.5°C	48 h	Microaerophilic
L3	F + P	10 mL	Yes	Bolton			mCCDA	Non-selective blood agar	42°C, 37°C	48 h	Microaerophilic
L4	P	20 µL	No				mCCDA		37°C	48 h	Microaerophilic
L5	V	10 µL	Yes	Preston	41.5°C	48 h	mCCDA	CAT	41.5°C	48 h	Microaerophilic
L6	F	10 mL	Yes	Bolton	41.5°C	48 h	mCCDA		41.5°	48 h	Microaerophilic
L7	P	100 µL	No				CUP		37°C	72 h	Microaerophilic
L8	P	10 µL	No				mCCDA		42°C	48 h	Microaerophilic
L9	F + V	NA*	NA*	NA*	NA*	NA*	NA*	NA*	NA*	NA*	NA*
L10	F	10 mL	Yes	Preston	41.5°C	24 h	mCCDA	Butzler	41.5°C	48 h	Microaerophilic
L11	V	10 mL	Yes	Preston	41.5°C	24 h	mCCDA	Butzler	41.5°C	48 h	Microaerophilic
L12	P	10 µL	Yes				Nonselective plate with cefuroxime***		42°C	48 h	Microaerophilic
L13	P	10 µL	No								
L14	F	10 µL	Yes	Preston	41°C	24 h	mCCDA		41°C	48 h	Microaerophilic
L15	F	NA*	NA*	NA*	NA*	NA*	NA*	NA*	NA*	NA*	NA*

V=animal health sector, F=food safety, P=public health; *Analysis not performed, **A wet swab from the samples was streaked onto selective media，*** Culture of positive PCR samples only

CAT: Cefoperazone amphotericin teicoplanin; mCCDA: Modified charcoal cephoperazone deoxycholate agar

Appendix table 2. Species identification of *Campylobacter* spp.

Lab code	Sector	Species identification	If PCR, reference	Comments
L1	F + V	PCR	Multiplex PCR (Ugarte-Ruiz et al. 2012)	If suspected colonies were found, streaked them onto blood agar 37ºC for 48 h following microaerobic conditions
L2	F + V	MALDI-TOF		
L3	F + P	PCR	Multiplex PCR (Denis et al. 1999)	
L4	P	MALDI-TOF		
L5	V	MALDI-TOF		
L6	F	Biochemical tests		From mCCDA to Blood Agar for confirmation.
L7	P	MALDI-TOF, WGS		
L8	P	Biochemical tests, MALDI-TOF, Microscopy		
L9	F + V	NA*	NA*	
L10	F	Biochemical tests, PCR	Multiplex PCR (Wang et al. 2002)	ISO 10272-1:2017
L11	V	MALDI-TOF	ISO 10272-1	
L12	P	MALDI-TOF, PCR	In-house PCR, target genes ceuE (*C. coli*) and mapA (*C. jejuni*)	
L13	P	MALDI-TOF, PCR	BD MAX system	
L14	F	WGS		
L15	F	NA*	NA*	

V=animal health sector, F=food safety, P=public health; *Analysis not performed

mCCDA: Modified charcoal cephoperazone deoxycholate agar

Ugarte-Ruiz M, Gómez-Barrero S, Porrero MC, Alvarez J, García M, Comerón MC, Wassenaar TM, Domínguez L. Evaluation of four protocols for the detection and isolation of thermophilic *Campylobacter* from different matrices. J Appl Microbiol. 2012 Jul;113(1):200-8. doi: 10.1111/j.1365-2672.2012.05323.x.

Denis M, Soumet C, Rivoal K, Ermel G, Blivet D, Salvat G, Colin P. Development of a m-PCR assay for simultaneous identification of *Campylobacter jejuni* and *C. coli*. Lett Appl Microbiol. 1999 Dec;29(6):406-10. doi: 10.1046/j.1472-765x.1999.00658.x.

Wang G, Clark CG, Taylor TM, Pucknell C, Barton C, Price L, Woodward DL, Rodgers FG. 2002. Colony multiplex PCR assay for identification and differentiation of *Campylobacter jejuni*, *C. coli*, *C. lari*, *C. upsaliensis*, and *C. fetus* subsp. *fetus*. J Clin Microbiol. 40(12):4744-4747. doi: 10.1128/JCM.40.12.4744-4747.2002.

ISO 10272-1:2017 Microbiology of the food chain — Horizontal method for detection and enumeration of *Campylobacter* spp. — Part 1: Detection method.

Appendix table 3. Sample size, enrichment and selective medium used for detection of *Salmonella* spp.

Lab code	Sector	Sample size	Pre-enrichment				Enrichment				Selective medium			
			Yes/No	Medium	Temperature	Period	Yes/No	Medium	Temperature	Period	Medium1	Medium2	Temperature	Period
L1	F + V	10 mL	Yes	BPW	37°C	16-20 h	Yes	MSRV	41.5°C	24 h±3 up to 48h±3	XLD	SMID2	37°C	24 h±3
L2	F + V	10 mL	Yes	BPW	37°C	24 h	Yes	MSRV	41.5°C	24 h	XLD		37°C	24 h
L3	F + P	25 mL	Yes	BPW			Yes	RV, MKTTn	42°C (RV)/ 37°C (MKTTn)	24 h	XLD	Chrom-agar	37°C	
L4	P	20 µL	No				Yes	Selenite	37°C	24 h	Red agar		37°C	24 h
L5	V	25 mL	Yes	BPW	37°C	24 h	Yes	MSRV	41.5°C	24 h	XLD	BGA	37°C	24 h
L6	F	10 mL	Yes	BPW	37°C	24 h	Yes	RV	42°C	24 h	XLD	BGA		
L7	P	100 µL	No				No				Basic agar	DC agar	37°C	24 h
L8	P	10 µL	No				Yes	RV	42°C	24 h	XLD		35°C	24 h
L9	F + V	10 mL	Yes	BPW	37°C	16-20 h	Yes	MSRV	41.5°C	24 h±3	XLD, BGA, novo	Rambach	37°C	24 h±3
L10	F	25 mL	Yes	BPW	37°C	24 h	Yes	MSRV, MKTTn	41.5°C	24 h	XLD	XLT-4	37°C	24 h
L11	V	10 mL	Yes	BPW	37°C	24 h	Yes	MSRV	41.5°C	24 h	XLD	BGA	37°C	24h
L12	P	10 µL	No				Yes	RV	40°C		XLD			
L13	P	10 µL	No				No							
L14	F	10 µL	No				Yes	MSRV	41°C	24 h	XLD	BGA	37°C	24 h
L15	F	10 mL	Yes	BPW	37°C	24 h	Yes	MSRV, MKTTn	41.5°C	24 h	XLD	BGA	37°C	24 h

V=animal health sector, F=food safety, P=public health

BGA: Brilliant green agar; BPW: Buffered peptone water; MKTTn: Muller-Kauffmann tetrathionate/novobiocin broth; MSRV: Modified semi-solid Rappaport-Vassiliadis enrichment media; RV: Rappaport-Vassiliadis; XLD: Xylose lysine deoxycholate agar; XLT-4: Xylose lysine tergitol-4

Appendix table 4. Species identification and determination of serovar of *Salmonella* spp.

Lab code	Sector	Species identification	Serotyping	Comments
L1	F + V	Biochemical tests	Classical slide agglutination	ISO 6579 (primary production protocol). Confirmation according to ISO 6579-1:2017
L2	F + V	ISO 6579-1:2017-04	ISO 6579-1:2017-04	EN ISO 6579-1:2017-04
L3	F + P		Classical slide agglutination	0,1 ml of pre-enrichment + 10 ml of RVS and 1 ml of pre-enrichment + 10 ml of MKTTn (selective enrichment)
L4	P	Biochemical tests, MALDI-TOF	WGS, classical slide agglutination	
L5	V	Biochemical tests, MALDI-TOF	Classical slide agglutination	
L6	F	Biochemical tests	Classical slide agglutination	Pre-enrichment with BPW for 18-20 h in 37°C. 100 ul transfer to RVS-broth and Incubation for 24 h in a in 42°C water bath. From RVS a loopful is streaked on agar BriS and XLD.
L7	P	MALDI-TOF, WGS	WGS, classical slide agglutination	
L8	P	MALDI-TOF	Classical slide agglutination	Day one: XLD agar (24 h in 35°C) and Rappaport enrichment broth (24 h in 42°C). Day two: cultivation to XLD from Rappaport broth (24 h in 35°C)
L9	F + V		Classical slide agglutination	EN-ISO 6579-1:2017
L10	F	Biochemical tests	Classical slide agglutination	NF U 47-100
L11	V	MALDI-TOF		
L12	P	MALDI-TOF, PCR		In-house PCR
L13	P	PCR		BD MAX system
L14	F	WGS		
L15	F	Biochemical tests, WGS	WGS, classical slide agglutination	EN-ISO 6579-1:2017

V=animal health sector, F=food safety, P=public health

BPW: Buffered peptone water; BriS: Brilliance *Salmonella*-agar; MKTTn: Muller-Kauffmann tetrathionate/novobiocin broth; RVS: Rappaport-Vassiliadis Soy peptone broth; XLD: Xylose lysine deoxycholate agar

ISO 6579-1:2017 Microbiology of the food chain — Horizontal method for the detection, enumeration and serotyping of *Salmonella* — Part 1: Detection of *Salmonella* spp.

AFNOR NF U47-100. Standard Methods of analysis in animal health - Search by isolation and identification of any specified serovar or serovar(s) of *Salmonella* in the environment of animal production, Paris.

Appendix table 5. Sample size, enrichment and selective medium used for detection of *Yersinia enterocolitica*.

Lab code	Sector	Sample size	Pre-enrichment				Enrichment				Selective medium					
			Yes/No	Medium	Temperature	Period	Yes/No	Medium	Temperature	Period	Medium1	Temperature	Period	Medium2	Temperature	Period
L1	F + V	10 mL	Yes	PSB	25°C	5 days	Yes	ITC	25°C	48 h	YER	30°C	48 h	SSDC	30°C	48 h
L2	F + V	1 mL	Yes	PSB	5°C	14 d	Yes				CIN	29°C	24 h			
L3	F + P	25 mL	No				Yes	PSB	25°C	48 h	CIN	30°C	48h	Chromogenic Yersinia agar	30°C	48 h
L4	P	20 µL	No				No				CIN			SSI Diagnostica Red agar plate	37°C	24 h
L5	V	NA*	NA*	NA*	NA*	NA*	NA*	NA*	NA*	NA*	NA*	NA*	NA*	NA*	NA*	NA*
L6	F	10 mL	Yes				Yes	PSB	25°C	3 h	CIN	30°C	24 h			
L7	P	100 µL	No				No				Basic agar	37°C	24 h	DC agar	37°C	24 h
L8	P	10 µL	No				No				CIN	30°C (day1) RT (day2)	24 h + 24 h			
L9	F + V	NA*	NA*	NA*	NA*	NA*	NA*	NA*	NA*	NA*	NA*	NA*	NA*	NA*	NA*	NA*
L10	F	25 mL	No				Yes	PSB/ITC	25°C	48 h	CIN	30°C	24 h			
L11	V	10 mL	No				Yes	ITC	22°C	48 h	CIN	30°C	48 h			
L12	P	10 µL	No				No				CIN	30°C	24-48 h			
L13	P	10 µL	No				No									
L14	F	NA*	NA*	NA*	NA*	NA*	NA*	NA*	NA*	NA*	NA*	NA*	NA*	NA*	NA*	NA*
L15	F	NA*	NA*	NA*	NA*	NA*	NA*	NA*	NA*	NA*	NA*	NA*	NA*	NA*	NA*	NA*

V=animal health sector, F=food safety, P=public health; *Analysis not performed

CIN: Cefsulodin irgasan novobiocin agar; ITC: Irgasan ticarcillin potassium chlorate broth; PSB: Peptone sorbitol bile salts broth; SSDC: *Salmonella*-*Shigella*-desoxycholate-calcium chloride

Appendix table 6. Species identification and determination of biotype of *Yersinia enterocolitica*.

Lab code	Sector	Species identification	Comments
L1	F + V	Biochemical tests, MALDI-TOF	Direct plating: 1 ml of the sample was streaked onto three plates of agar YER. These plates were incubated at 30°C for 48 h. Suspected colonies were streaked onto blood agar being incubated at 30°C for 48 h.<br>Enrichment on PSB (peptone, sorbitol and bile salts): 10 ml of the sample were diluted onto 90 ml of PSB and incubated at 25°C during 5 days. After this incubation, 25 µl was streaked onto agar YER being incubated at 30ºC for 48 h. Suspected colonies were streaked onto blood agar being incubated at 30ºC for 48 h.<br>Enrichment on ITC: 10 ml of the sample diluted on PSB (before being incubated) were diluted onto 90 ml of ITC (1/100 form the original sample) and incubated at 25°C during 48h. After this incubation, 25 µl were streaked onto agar SSDC being incubated at 30°C for 48 h. Suspected colonies were streaked onto blood agar being incubated at 30ºC for 48 h.
L2	F + V	MALDI-TOF	Pre-enrichment: 10 ml of sample added into 90ml PSB broth, incubated at 5°C (14 days), 10 µl subculture suspended<br>in solution 0.25% KOH in 0.5% NaCl, mixed and one loopful (1 µL loop) was streaked over the surface of CIN agar (29°C, 24h)
L3	F + P	Biochemical tests, PCR	ISO 18867 (Real time PCR for *Y. enterocolitica* - target ail gene); real time PCR for *Y. enterocolitica- ystA* and *ystB* genes
L4	P	MALDI-TOF, Bio/serotyping	
L5	V	NA*	
L6	F	Biochemical tests, Bio-/serotyping	PSB (for 3 h) at 25°C. PSB_> 10 µL and 100 µL onto CIN agar. Incubation at 30°C for 24h. PSB is incubated at 4°C.<br>Day 2: from CIN to BS. Day 8: 100 µL PSB to 10 mL MRB incubated at 25°C for 4 days. Day 12: MRB to CIN and day 13 to BS.
L7	P	MALDI-TOF, WGS	
L8	P	MALDI-TOF, esculin	
L9	F + V	NA*	
L10	F	Biochemical tests	First, streaking of typical colonies (from CIN) on YeCM to differentiate non-pathogenic Ye (BT1A) from pathogenic YE, then biotyping with ISO 10273:2017
L11	V	MALDI-TOF, esculin	
L12	P	MALDI-TOF, esculin, PCR	In-house PCR target ail gene for *Y. enterocolitica* and *Y. pesudotuberculosis*.
L13	P	PCR	BD MAX system
L14	F	NA*	

L15	F	NA*

V=animal health sector, F=food safety, P=public health; *Analysis not performed

BS: Bromthymol blue saccharose; CIN: Cefsulodin irgasan novobiocin agar; ITC: Irgasan ticarcillin potassium chlorate broth; MRB: Modified Rappaport broth; PSB: Peptone sorbitol bile salts broth; SSDC: *Salmonella*-*Shigella*-desoxycholate-calcium chloride; YeCM: *Y. enterocolitica* chromogenic medium

ISO/TS 18867:2015 Microbiology of the food chain — Polymerase chain reaction (PCR) for the detection of food-borne pathogens — Detection of pathogenic *Yersinia enterocolitica* and *Yersinia pseudotuberculosis*

ISO 10273:2017 Microbiology of the food chain — Horizontal method for the detection of pathogenic *Yersinia enterocolitica*
